# RNAseq analysis of *Aspergillus fumigatus* in blood reveals a just wait and see resting stage behavior

**DOI:** 10.1186/s12864-015-1853-1

**Published:** 2015-08-27

**Authors:** Henriette Irmer, Sonia Tarazona, Christoph Sasse, Patrick Olbermann, Jürgen Loeffler, Sven Krappmann, Ana Conesa, Gerhard H. Braus

**Affiliations:** Institut für Mikrobiologie und Genetik, Georg-August-Universität Göttingen, Grisebachstraße 8, D-37077 Göttingen, Germany; Genomics of Gene Expression Lab, Prince Felipe Research Center, Av. Eduardo Primo Yufera 3, 46012 Valencia, Spain; Department of Microbiology and Cell Science, Institute for Food and Agricultura Sciences, University of Florida at Gainesville, Gainesville, FL USA; Research Center for Infectious Diseases, Julius-Maximilians University Würzburg, Würzburg, Germany; Laboratory WÜ4i, Medical Clinic and Policlinic II, University Clinic Würzburg, Würzburg, Germany; Mikrobiologisches Institut – Klinische Mikrobiologie, Immunologie und Hygiene, Universitätsklinik Erlangen and Friedrich-Alexander-Universität Erlangen-Nürnberg, Erlangen, Germany

**Keywords:** *Aspergillus fumigatus*, mRNA-Seq, Transcriptome, Human pathogenic fungi, Iron homeostasis, Secondary metabolite gene cluster, Detoxification

## Abstract

**Background:**

Invasive aspergillosis is started after germination of *Aspergillus fumigatus* conidia that are inhaled by susceptible individuals. Fungal hyphae can grow in the lung through the epithelial tissue and disseminate hematogenously to invade into other organs. Low fungaemia indicates that fungal elements do not reside in the bloodstream for long.

**Results:**

We analyzed whether blood represents a hostile environment to which the physiology of *A. fumigatus* has to adapt. An *in vitro* model of *A. fumigatus* infection was established by incubating mycelium in blood. Our model allowed to discern the changes of the gene expression profile of *A. fumigatus* at various stages of the infection. The majority of described virulence factors that are connected to pulmonary infections appeared not to be activated during the blood phase. Three active processes were identified that presumably help the fungus to survive the blood environment in an advanced phase of the infection: iron homeostasis, secondary metabolism, and the formation of detoxifying enzymes.

**Conclusions:**

We propose that *A. fumigatus* is hardly able to propagate in blood. After an early stage of sensing the environment, virtually all uptake mechanisms and energy-consuming metabolic pathways are shut-down. The fungus appears to adapt by trans-differentiation into a resting mycelial stage. This might reflect the harsh conditions in blood where *A. fumigatus* cannot take up sufficient nutrients to establish self-defense mechanisms combined with significant growth.

**Electronic supplementary material:**

The online version of this article (doi10.1186/s12864-015-1853-1) contains supplementary material, which is available to authorized users.

## Background

*Aspergillus fumigatus* is one of the most frequent causes of fungal infections of immunocompromised patients, with mortality rates of over 50 % [1–3]. The fungus can invade susceptible patients through the lung and cause invasive pulmonary aspergillosis (IPA) as the most severe form. The survival of *A. fumigatus* in the host is probably a multifactorial trait and not caused by a specific virulence strategy [4, 5]. Indeed, the fungus can survive in competitive environments, a likely prerequisite of its pathogenicity and worldwide ubiquitous distribution in decaying organic material [6]. During IPA, fungal hyphae penetrate the epithelial tissue of the lung, causing angioinvasion, and eventually disseminate via the bloodstream to colonize other organs [7, 8]. It is generally assumed that the hyphae do not reside in the bloodstream at significant amounts [9].

Several conditions and determinants that play a role during pulmonary infection have been identified, including hypoxia, thermotolerance, iron acquisition, and secondary metabolite production [10–14]. The biosynthesis of amino acids also appears important because the cross-pathway control transcriptional activator CpcA is crucial for virulence [15] and several auxotrophic mutants are avirulent [16, 17].

In order to better understand the infection process it is crucial to establish suitable animal models that reflect the infection in humans. An overview of infection models to study the virulence of *A. fumigatus* is available [18]. In most cases, the neutropenic or non-neutropenic murine model of pulmonary aspergillosis is used [19]. In these approaches, mice are treated for immunosuppression with chemotherapeutic agents and/or corticosteroids. The conidia as infectious propagules are either applied intranasally or intratracheally, or they are actively inhaled by the animals in an aerosol chamber. The mice develop respiratory distress and are sacrificed when developing severe symptoms to reduce suffering [20]. Importantly, this model mimics the dissemination process upon invasive aspergillosis (IA) only to a limited extent. Systemic IA is induced by injecting conidia into the tail vein of mice or after intranasal and intratracheal administration [21, 22], eventually resulting in colonization of the kidney. In a model of cerebral aspergillosis, conidia are injected intracerebrally into mice or rats [23, 24]. These models have proven very valuable for testing novel drugs, but since the lung is not affected in these models, the dissemination process in human IA is mimicked only partially [25].

Transcriptomic and proteomic approaches are well suitable to identify a wide spectrum of factors involved in infection. In theory, mouse models should allow to measure the expression of fungal genes during infection. However, it has remained a challenge to obtain sufficient fungal material for the analysis of RNA. Therefore only three approaches with material from infection models had been published so far [26–28]. By microarray-based hybridizations, the transcriptome of *A. fumigatus* was determined at an early stage of infection that is after germination of the conidia in the lung. It was observed that the fungal transcriptome displayed changes that imply iron limitation, alkaline stress, and nutrient deprivation. To profile under isolated conditions and overcome the limited availability of material, *in vitro* approaches were carried out mimicking certain infectious environments. Transcriptome data were obtained from various experimental conditions like hypoxia, thermostress, oxidative stress, interaction with cells of the immune system and varying growth conditions [29–35]. Several transcriptome analyses dealt with recombinant strains deleted for known virulence factors with regulatory properties like MpkA, GprD/C and LaeA [36–38]. The latter showed that in a *ΔlaeA* background many clusters are differentially expressed that are likely to reflect changes affecting the biosynthesis of secondary metabolites. Gene expression profiles were also investigated during antimycotic treatment. For example, amphotericin B induced transcription profile changes affecting genes related to the ergosterol pathway, cell stress, cell wall, and transport [39], while voriconazole affected the ergosterol and cAMP PKS signaling-pathways [40]. These transcriptome data were mostly obtained using various microarray hybridization platforms and in some cases cDNA sequencing at high throughput (RNA-Seq). A comparison of several published transcriptome datasets has revealed that results from different RNA-Seq datasets are more comparable among each other than those arising from different microarray platforms [37]. Thus, RNA-Seq is currently the method of choice to monitor gene expression in any microorganism, such as *A. fumigatus*.

During aspergillosis, the shift from pulmonary colonization to that of other organs is rather transient. The dissemination process is difficult to mimic, despite it is an important stage of the infection because the colonization of organs relates to the high mortality during aspergillosis. Drugs that target this stage could be capable of blocking the dissemination and thus prevent rapid life threatening progression of the infection. Knowledge is scarce about the fungal gene expression changes to adapt to the harsh conditions within the bloodstream. The fungal burden in blood is generally too low to be detected by blood cultures. The human pathogenic fungus *Candida albicans* shows a similar dissemination via the bloodstream during infection. Upon incubation with blood *C. albicans* showed differential expression in genes involved in general stress response, antioxidative response, glyoxylate cycle and hyphal growth [41].

We established an *in vitro* model for the incubation of *A. fumigatus* in human blood in order to further determine how the fungus’ gene expression profile reacts to this specific and relevant condition. We have used RNA-Seq to determine the transcriptome of the fungus incubated in blood or in minimal medium as reference condition. Based on our results we propose that *A. fumigatus* cannot significantly grow in blood. Instead, after an early stage of sensing the environment, the fungus adapts by entering a resting mycelial stage, thereby shutting down virtually all uptake mechanisms and energy-consuming metabolic pathways. This reaction might reflect a condition in which *A. fumigatus* cannot take up sufficient nutrients and protect itself against the harsh conditions in blood at the same time.

## Results and discussion

### Investigating the transcriptome of *Aspergillus fumigatus* incubated in human blood

**Fig. 1:**
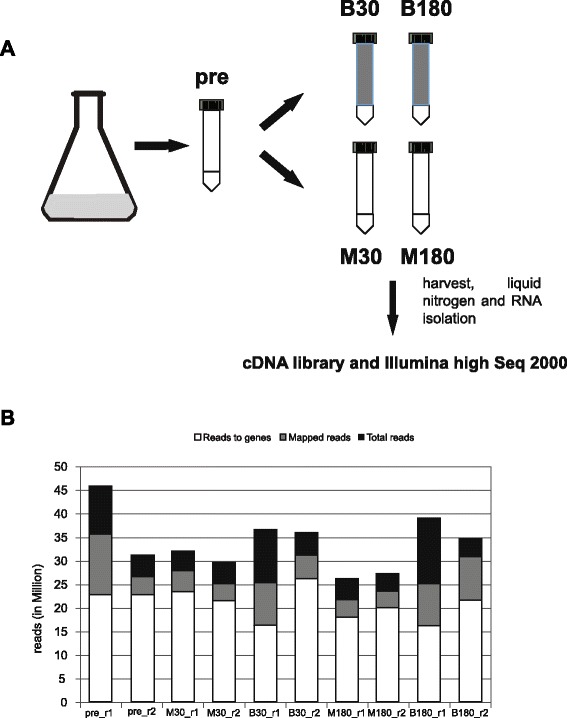
Schematic display of the *in vitro* blood model and mapping results. **a** For the *in vitro* model 2 ml mycelium from an overnight culture were aliquoted and, except for the pre value, incubated in either blood or minimal medium (10 ml) for 30 and 180 min at 37 °C. The complete mycelium was harvested immediately after the procedure and snap frozen in liquid nitrogen. RNA was isolated, cDNA libraries were generated and Illumina high Seq 2000 sequencing was performed. **b** Total number of reads in each sample (Total reads), number of reads aligned to the reference genome (Mapped reads), and number of mapped reads allocated to annotated genes (Reads to genes). These numbers are indicated by the top side of the corresponding bars

Invasive aspergillosis is established after infection of lung tissue by the dissemination of mycelial fragments through the bloodstream. In order to determine transcriptional changes during the inoculation of *A. fumigatus* mycelium in human blood, we set up an *in vitro* assay. For this we used freshly drawn, heparinized blood from one individual and harvested total RNA at different experimental conditions. In a microarray pilot experiment we monitored different time points. Based on these pilot experiments we decided to select five conditions with two biological replicates (r1 and r2) for further analysis including the pre-culture (pre) prior to the change of medium and a comparison between growth after half an hour or three hours in either blood (B) or defined minimal medium (M): pre, M30, B30, M180 and B180 (Fig. 1a). cDNA libraries were generated and subsequently sequenced on an IlluminaHiSeq 2000 platform by GATC biotech (Konstanz, Germany). The statistical method used for differential expression was NOISeq [42]. A control lacking heparin was omitted since previous experiments in *C. albicans* did not show significant influence of heparin on fungal gene regulation [41].

### Analysis of differentially expressed genes

**Fig. 2:**
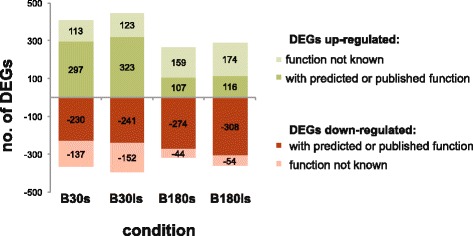
Number of differential expressed genes in blood culture. List for B30s and B180s contain differential expressed genes (DEGs) in blood under stringent conditions. Under these conditions DEGs have no or opposite differential expression in the corresponding minimal medium condition. B30ls and B180ls include differentially expressed genes under less stringent conditions, that also show regulation in minimal medium but to a lesser or higher extend than in blood. For details see text. Positive values are up-regulated DEGs and negative values corresponds to the number of down-regulated DEGs. In B180 up-regulated DEGs for few genes the function was characterized or predicted

The transcriptome of the fungus was monitored in an earlier stage (30 min) and at a later stage (180 min) in the course of incubation in human blood or defined culture medium. The list of differentially expressed genes (DEGs) exclusively expressed in blood (B30s and B180s) was obtained by relating RNA levels from the blood condition (B30, B180) and medium condition (M30, M180) to the initial 16 h liquid culture (pre). The genes that displayed similar differential expression in both were subtracted. Hence, those genes that showed differential expression only in blood are represented in the list (B30s and B180s, s denotes stringent). Table 1 lists the top range of differentially expressed genes (cutoff >2/<−2). A further, less stringent list was obtained by additionally including those genes that showed differential expression in both medium and blood but to a lesser extent in medium when compared to the blood condition (B30ls and B180ls). A gene was included in the less stringent list when the difference of the M value (log2-fold change) was more than 0.8 in the conditions M30-pre to B30-pre (M180-pre to B180-pre) (see Additional file 1: Table S2 sheet B and C). Additional file 1: Table S2 sheet A lists the differentially expressed genes (DEGs B30s, B180s and B30ls, B180ls) used for further analysis. The number of stringently differentially regulated genes in B30s was 777 (410 up and 367 down regulated) whereas in B180s this number was reduced to 584 genes (266 up and 318 down regulated). Similarly, 839 less stringently controlled genes of B30ls (446 up and 393 down regulated) corresponded to 652 genes in the B180ls list (290 up and 362 down regulated) (Fig. 2). This corresponds to approximately 8 % for B30 and 6 % for B180 of the expressed genes. From the up-regulated DEGs at B180s and B180ls only 40 % of the genes were functionally annotated.

**Table 1:** List of the furthest differentially expressed genes in blood after 30 min (B30s) and after 180 min (B180s)

Afua	Annotation	B30s	B180s
AFUA_8G01260	hypothetical protein	−2.66	−2.05
AFUA_4G08420	hypothetical protein	−2.25	
AFUA_5G02700	MFS multidrug transporter	−2.17	−1.51
AFUA_6G13380	arrestin (or S-antigen), N-terminal domain	−2.17	−1.12
AFUA_4G09190	S-adenosyl-methionine-sterol-C-	−2.15	
AFUA_2G11260	3-isopropylmalate dehydratase	−2.05	
AFUA_4G08710	short chain dehydrogenase	−2.00	
AFUA_7G00970	GPI anchored serine-threonine rich protein	2.14	2.37
AFUA_6G12920	C2H2 finger domain protein	2.14	
AFUA_2G05560	exonuclease	2.19	
AFUA_3G10690	calcium-translocating P-type	2.19	
AFUA_3G15080	hypothetical protein	2.20	
AFUA_1G13070	U3 small nucleolar ribonucleoprotein protein	2.21	
AFUA_6G14260	U3 small nucleolar ribonucleoprotein protein	2.23	
AFUA_3G06380	exosome-associated family protein	2.24	
AFUA_4G14180	hypothetical protein	2.25	
AFUA_2G02500	hypothetical protein	2.26	1.60
AFUA_5G04010	tRNA-splicing endonuclease subunit Sen2	2.32	
AFUA_2G02970	hypothetical protein	2.34	
AFUA_6G03750	amidophosphoribosyltransferase	2.38	
AFUA_8G01850	phosphate-repressible phosphate permease	2.43	
AFUA_8G05720	DUF567 domain protein	2.44	
AFUA_4G14190	hypothetical protein	2.46	
AFUA_7G06030	alpha-ketoglutarate-dependent taurine	2.49	
AFUA_4G12940	hypothetical protein	2.78	1.42
AFUA_5G08680	mitochondrial GTPase (YlqF)	2.93	
AFUA_4G14160	hypothetical protein	3.06	3.18
AFUA_7G00340	metallo-beta-lactamase domain protein	3.10	
AFUA_8G07170	hypothetical protein	3.13	
AFUA_2G17240	C2H2 finger domain protein	3.25	3.32
AFUA_2G08180	flotillin domain protein	3.55	
AFUA_6G04690	hypothetical protein	3.67	
AFUA_3G03050	RNAse III	3.68	3.88
AFUA_3G01196	hypothetical protein	3.78	2.54
AFUA_8G04380	hypothetical protein	4.55	3.38
AFUA_8G05910	pectate lyase	4.58	3.91
AFUA_4G14170	hypothetical protein	4.89	3.75
AFUA_5G14410	cysteine dioxygenase	5.96	7.05
AFUA_7G05660	translation elongation factor eEF-3		−2.36
AFUA_5G05590	aspartokinase		−2.37
AFUA_1G10130	adenosylhomocysteinase		−2.09
AFUA_5G11340	translation initiation factor eIF-2B alpha		−2.01
AFUA_8G05960	integral membrane protein		2.13
AFUA_2G05720	hypothetical protein		2.14
AFUA_5G02300	peroxidase		2.16
AFUA_8G01630	pyridine nucleotide-disulphide oxidoreductase		2.38
AFUA_4G00750	hypothetical protein		2.46
AFUA_5G13830	alpha-galactosidase		2.50
AFUA_8G05950	hypothetical protein		2.91
AFUA_7G06330	hypothetical protein		3.03

Genes with highest levels of differential expression are shown with a cutoff at > 2 or <−2. The number represents the log2-ratio or fold change for positive (up-regulated) and negative (down-regulated) genes

**Fig. 3:**
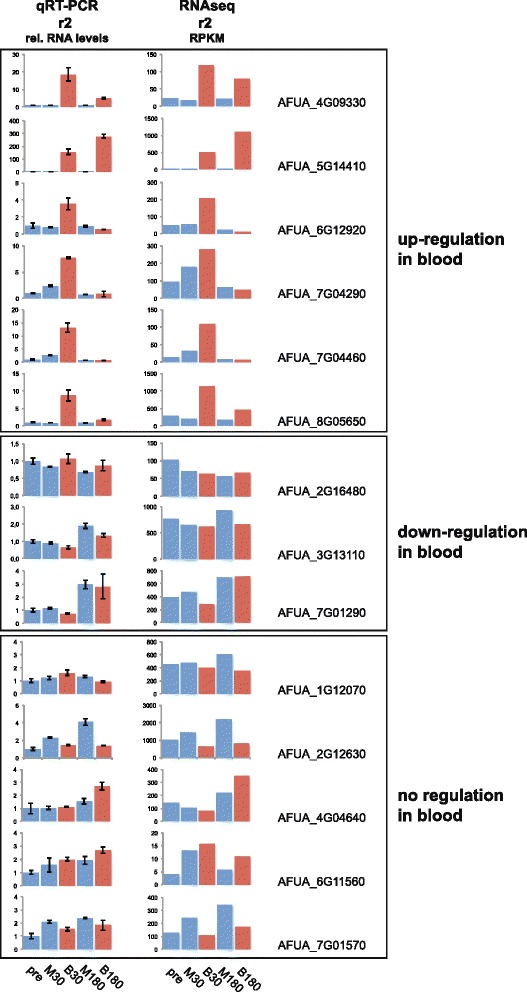
Relative mRNA level regulation is comparable in most cases with RNAseq data. Comparison of relative mRNA expression from biological replicate r2 using quantitative RT-PCR (qRT-PCR) and RNAseq of various genes show that both methods yield similar results in most cases. Shown are representative genes that were identified as up-regulated in blood, down-regulated in blood and not regulated. Note that all graphs have different scaling and show relative expression levels for qRT-PCR and RPKM values for RNAseq data

Regulation patterns of transcriptome sequencing were in accordance to quantitative real time (qRT) PCR experiments applied to selected genes in order to verify the expression data obtained by the RNA-Seq approach (Additional file 2: Table S1). Genes were chosen from different categories: up-regulated in blood, not-regulated, or down-regulated in blood. The obtained relative expression levels of the biological control r2 showed in most cases equivalent regulation when compared to the RPKM values of transcriptome sequencing data (Fig. 3). Genes especially discussed in the following paragraphs were tested with qRT-PCR on both biological replicates r1 and r2 and compared to the corrected RPKM from RNA-Seq (Additional file 3: Figure S1 A,B and C). Overall the data obtained by qRT-PCR were highly consistent with the transcriptome sequencing data.

### Functional analysis

**Fig. 4:**
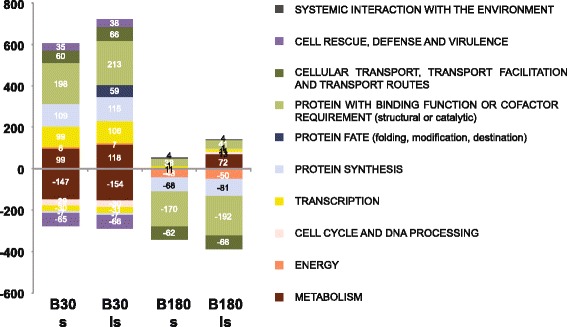
Functional enrichment analysis with FunCat. The functional categories of the first level that are significantly enriched upon differentially expressed genes from B30s, B30ls, B180s and B180ls. Few categories were found that are up-regulated in B180s and B180ls. *P*-value cutoff was 0.005

We have employed the functional catalogue (FunCat) and the gene ontology (GO) for the functional enrichment analysis. The goal of this analysis was to identify functionally enriched terms in the lists of DEGs previously described. We used the FungiFun platform for the B30s, B180s, and the less stringent B30ls, and B180ls lists [43]. The results are presented for the analyses of the stringent conditions, while data for the less stringent conditions will be given only if they principally differ from the stringent conditions. The data are listed in Additional file 4: Table S3 and summarized in Fig. 4. We used the FunCat categories as the overall structure of the presentation and additionally included relevant GO term categories. Interestingly, the FunCat and GO analyses displayed only minimal differences between the stringent and the less stringent conditions, and only in few cases additional categories were found to be enriched (Fig. 4). From relevant categories several associated DEGs were further investigated using the *Aspergillus* Genome Database and links within to get a better understanding about the function of the gene in the category [44]. In the following sections the most striking categories and corresponding DEGs are described and summarized in Table 2. In Fig. 4 it is apparent that the majority of enriched categories are linked at 30 min to up-regulated and at 180 min to down-regulated genes. In order to get a better overview about the regulation in blood we describe in the following sections four regulation patterns of the functional enriched categories: early regulation with the majority of up-regulated genes (at 30 min), early up-regulation and subsequent late down-regulation, solely late down-regulation (at 180 min) and late up-regulation. Some of the identified DEGs were also tested with qRT-PCR to verify their regulation (Fig. 3 and Additional file 3: Figure S1AB and C).

**Table 2:** Functional categorization of selected genes

Number	Name	Known description, putative function or ortholog function
B30 up-regulation		
Secondary metabolism		
AFUA_3G00840		FAD-dependent oxygenase, required for asperfuranone biosynthesis in *A. nidulans* (afoF) FAD-dependant oxygenase; secreted protein
AFUA_3G12910	hasC	O-methyltransferase GliM-like, orthologous member of the “has” secondary metabolite biosynthetic gene cluster of strain A1163. [67]
AFUA_3G12930	hasE	dimethylallyl tryptophan synthase SirD-like, orthologous member of the “has” secondary metabolite biosynthetic gene cluster of strain A1163. [67]
Transcriptional control		
AFUA_5G06190	steA	sexual development transcription factor, involved in sexual development
AFUA_5G05610		putative cell cycle control protein Cwf14/Bud31, ortholog(s) have role in cellular bud site selection
AFUA_1G12940	sakA	MAP kinase SakA, nitrogen sensing, is induced upon carbon and nitrogen starvation [52]
Transport		
AFUA_1G12620	aflT	MFS toxin efflux pump
AFUA_7G04290		GapA, putative amino acid transporter
AFUA_8G02200		proline permease, putative amino acid transporter
Stress response		
AFUA_5G06520		low-temperature viability protein ltv1, ortholog(s) have role in cellular response to oxidative stress, response to osmotic stress
AFUA_5G11230	rasA	RAS small monomeric GTPase RasA, involved in cell wall integrity
Chaperone and protein folding activity		
AFUA_2G02320		Hsp70 chaperone (BiP)
AFUA_4G03650		ribosome associated DnaJ chaperone Zuotin,
AFUA_8G03930	hscA	Hsp70 chaperone HscA, Yap1-dependent induction in response to hydrogen peroxide; hypoxia induced protein
AFUA_2G16290		DnaJ domain protein Mas5, ortholog(s) have endoplasmic reticulum localization
AFUA_4G12360		prefoldin subunit 1, has domain(s) with predicted unfolded protein binding activity, role in protein folding and prefoldin complex localization
Detoxification		
AFUA_4G12990	trr1	thioredoxin reductase Trr1/Trr2
AFUA_4G00930		CorA family metal ion transporter, Mg transport
AFUA_2G13080		Nudix/MutT family protein, ortholog(s) have hydrolase activity
AFUA_1G12620	aflT	MFS toxin efflux pump
Lipid acid and isoprenoid metabolism		
AFUA_8G04080	badA	betaine aldehyde dehydrogenase, involved in glycin betain biosynthesis [45]
AFUA_8G04090	codA	choline oxidase, involved in glycin betain biosynthesis [45]
B30 down-regulation		
Secondary metabolism:		
AFUA_1G10380	nrps1	nonribosomal peptide synthase Pes1, essential for fumigaclavine C biosynthesis [47]
AFUA_3G03350	sidE	nonribosomal peptide synthase SidE, involved in production of fumarylalanine [48]
Transcription		
AFUA_1G12332		jumonji family transcription factor, has domain(s) with predicted zinc ion binding activity
AFUA_1G13750		C2H2 transcription factor Rpn4, proteasomal regulator in *S. cervisiae*
AFUA_1G15230		C6 transcription factor, ortholog(s) have role in conidiophore development, conidium formation, hyphal growth
AFUA_1G16460		bZIP transcription factor (LziP), ortholog(s) have role in asexual sporulation, sexual sporulation resulting in formation of a cellular spore
AFUA_2G11780		CreA, C2H2 transcription factor
AFUA_2G13260	medA	MedA, transcriptional regulator of adherence, host cell interactions and virulence; required for wild-type levels of conidiation
AFUA_2G14110		sulfur metabolite repression control protein, ortholog(s) have protein binding, bridging, ubiquitin binding activity
AFUA_2G14680	flbB	Bzip developmental regulator; role in asexual development, gliotoxin production
AFUA_2G16230		C2H2 finger domain protein, has domain(s) with predicted DNA binding, zinc ion binding activity, role in transcription
AFUA_2G17220		C2H2 transcription factor (AmdX), ortholog(s) have sequence-specific DNA binding activity, role in regulation of transcription
AFUA_3G02000		farB like, C6 transcription factor Ctf1B-like, putatively involved in fatty acid degradation
AFUA_3G05760		C6 transcription factor (Fcr1)
AFUA_3G06250		RNA polymerase II mediator complex component, has domain(s) with predicted RNA polymerase II transcription cofactor activity
AFUA_3G11250	ace2	C2H2 transcription factor (Swi5), with a role in conidiophore development, pigment production, germination and virulence
AFUA_3G12940	hasF	C6 transcription factor, orthologous member of the “has” secondary metabolite biosynthetic gene cluster of strain A1163. [67]
AFUA_4G03960	farA	C6 transcription factor Ctf1A, with a role in beta oxidation of long-chain fatty acids
AFUA_6G12160		C6 transcription factor
Cell wall		
AFUA_6G06340	gfa1	glucosamine-fructose-6-phosphate transaminase, chitin production
AFUA_2G01170	gel1	1,3-beta-glucanosyltransferase, biosynthesis of fungal cell wall [91]
AFUA_2G05340	gel4	1,3-beta-glucanosyltransferase, GPI-anchored to the plasma membrane; cell wall maintanance
Resistance proteins		
AFUA_1G03200	mfsC	MFS transporter
AFUA_1G10370		MFS multidrug transporter
AFUA_1G10390	abcB	ABC multidrug transporter
AFUA_1G13800		MFS multidrug transporter
AFUA_3G02610		MFS transporter, has domain(s) with predicted role in transmembrane transport and integral to membrane localization
AFUA_5G00980		MFS multidrug transporter, putative transporter of small solutes
AFUA_5G02700		MFS multidrug transporter, putative transporter of small solutes
AFUA_7G01790	ssu1	MFS transporter, putative sulphite efflux pump
AFUA_2G14570		zinc/cadmium resistance protein, ortholog(s) have zinc ion transmembrane transporter activity, role in cellular zinc ion homeostasis
AFUA_6G02220		MFS toxin efflux pump
AFUA_8G02050		putative transporter of polysaccharides
Heat shock, chaperone activity		
AFUA_1G07440	hsp70	molecular chaperone Hsp70
AFUA_1G06020		DnaJ domain protein, has domain(s) with predicted heat shock protein binding, unfolded protein binding activity and role in protein folding
AFUA_1G11180	hsp78	heat shock protein/chaperonin HSP78
AFUA_1G15270		heat shock protein Hsp98/Hsp104/ClpA, ortholog(s) have cytosol, nuclear envelope localization
AFUA_3G14540	hsp30	heat shock protein Hsp30/Hsp42, putative 30-kDa heat shock protein
AFUA_4G12850	clxA	calnexin, ER membrane chaperone involved in folding of glycoproteins directed to the secretory pathway
AFUA_5G07340		DnaJ domain protein Psi, ortholog(s) have cytosol, nucleus localization, involved in protein folding and translational initiation
AFUA_6G06470		heat shock protein Hsp30-like, putative integral plasma membrane heat shock protein
B30 up-regulation and 180 min down-regulation		
Protein/ peptide degradation and modification		
AFUA_5G06060	skpA	sulfur metabolism regulator SkpA, part of SCF ubiquitin ligase complex
AFUA_1G04040	ubiA	ubiquitin
AFUA_5G04000		protein involved in proteasomal and 4S ribosomal subunit biogenesis
Peptidases		
AFUA_3G05450		glutamate carboxypeptidase
AFUA_6G04510		O-sialoglycoprotein endopeptidase, ortholog(s) have chromatin DNA binding activity and role in positive regulation of transcription from RNA polymerase II promoter
AFUA_6G07330		methionine aminopeptidase, type I, orthologs have functions in protein processing
Glycosylation		
AFUA_6G12360		class I alpha-mannosidase 1A, has domain(s) with predicted calcium ion binding, mannosyl-oligosaccharide 1,2-alpha-mannosidase activity
AFUA_4G10070		class I alpha-mannosidase, has domain(s) with predicted calcium ion binding, mannosyl-oligosaccharide 1,2-alpha-mannosidase activity
AFUA_1G12630		mannosylphosphate transferase (Mnn4), putative phosphorylcholine metabolism
AFUA_4G10750		alpha-1,6-mannosyltransferase subunit, Glycan biosynthesis
AFUA_5G05740	gmtA	Golgi GDP-mannose transporter, involved in biosynthesis of the cell wall polysaccharide galactomannan
Phosphorylation		
AFUA_6G12820	mpkB	MAP kinase MpkB, putative mitogen-activated protein kinase
AFUA_5G11730		RIO1 family protein kinase, ortholog(s) have nucleocytoplasmic transporter activity, protein kinase activity, role in maturation of SSU-rRNA
AFUA_1G12940	sakA	MAP kinase SakA, nitrogen sensing, is induced upon carbon and nitrogen starvation [52]
AFUA_1G09170		protein kinase activator (Mob2), ortholog(s) have kinase binding activity and role in conidiophore development, hyphal growth, regulation of kinase activity
AFUA_1G16000		serine/threonine protein kinase, has domain(s) with predicted ATP binding, protein tyrosine kinase activity and role in protein phosphorylation
AFUA_2G13680		calcium/calmodulin-dependent protein kinase, orthologs have a function in stress response
AFUA_2G05490		HEAT repeat protein, ortholog(s) have deoxyhypusine monooxygenase activity
AFUA_3G12490		protein arginine methyltransferase RmtB, ortholog(s) have histone-arginine N-methyltransferase activity
AFUA_4G13400		glycosyltransferase family 28, ortholog(s) have N-acetylglucosaminyldiphosphodolichol N-acetylglucosaminyltransferase activity
AFUA_5G01740		deoxyhypusine synthase, putatively first step in hypusine biosynthesis
180 min down-regulation		
ATP binding (GO:0005524)		
AFUA_1G02570		aspartyl-tRNA synthetase, cytoplasmic, aspartyl-tRNA synthetase; calcium downregulated
AFUA_1G05620		phenylalanyl-tRNA synthetase, beta subunit, ortholog(s) have phenylalanine-tRNA ligase activity
AFUA_1G05800	mkk2	MAP kinase kinase (Mkk2), putative mitogen-activated protein kinase kinase (MAPKK); essential for cell wall integrity signaling; calcium induced
AFUA_1G09010		methionyl-tRNA synthetase, ortholog(s) have cytosol localization
AFUA_1G10310		RNase L inhibitor of the ABC superfamily, ATPase, Rnase L inhibitor, orthologs involved in ribosome protection
AFUA_1G10630		S-adenosylmethionine synthetase, putative S-adenosylmethionine synthetase; involved in methylation processes, biosynthesis of biotin and polyamines
AFUA_2G02320		Hsp70 chaperone (BiP)
AFUA_2G02590		aspartyl-tRNA synthetase Dps1
AFUA_2G03580		phenylalanyl-tRNA synthetase beta chain, ortholog(s) have phenylalanine-tRNA ligase activity
AFUA_2G04620	bipA	Hsp70 chaperone BiP/Kar2
AFUA_2G05650		cytoplasmic asparaginyl-tRNA synthetase, ortholog(s) have asparagine-tRNA ligase activity, role in asparaginyl-tRNA aminoacylation and cytosol localization
AFUA_2G06110		chromatin remodeling and histone, ortholog(s) have role in chromatin silencing at centromere
AFUA_2G08670		acetyl-CoA carboxylase, ortholog(s) have acetyl-CoA carboxylase activity, biotin carboxylase activity and role in long-chain fatty acid biosynthetic
AFUA_2G09290	hsp60	antigenic mitochondrial protein HSP60, involved in heat shock response
AFUA_2G13140	ime2	meiosis induction protein kinase, a serine/threonine protein kinase involved in mycotoxin production and sex. development
AFUA_2G14030		arginyl-tRNA synthetase, ortholog(s) have arginine-tRNA ligase activity, role in arginyl-tRNA aminoacylation and cytosol, mitochondrion localization
AFUA_3G05430		DEAD-box RNA helicase Dhh1/Vad1, ortholog(s) have translation regulator activity, nucleic acid binding activity
AFUA_3G08650		C1 tetrahydrofolate synthase
AFUA_3G10300		putative galactokinase with a role in galactose catabolism
AFUA_3G10530		protein serine/threonine kinase (Ran1), orthologs have negative regulation of transcription/ development-meiosis
AFUA_3G13030		t-complex protein 1, gamma subunit (Cct3), ortholog(s) have unfolded protein binding activity, role in cellular response to drug, protein folding
AFUA_4G07660	ded1	ATP dependent RNA helicase (Dbp1), ATP-dependent RNA helicase, cell cycle affected in *S. pombe*
AFUA_4G08060		tetrahydrofolylpolyglutamate synthase (Met7), role in one-carbon metabolic process, regulation of DNA methylation and cytosol, mitochondrion
AFUA_4G13700		threonyl-tRNA synthetase, putative tRNA charging
AFUA_5G05490		seryl-tRNA synthetase,
AFUA_5G05960		serine/threonine protein kinase, involved in salt tolerance in *S. cerevisiae*
AFUA_5G07020		ribosome biogenesis ABC transporter Arb1
AFUA_5G09610		cysteinyl-tRNA synthetase, putative tRNA charging
AFUA_5G10550		ATP synthase F1, beta subunit, orthologs are required for synthesis of ATP, but are non-essential
AFUA_6G04730		bifunctional purine biosynthetic protein Ade1, ortholog(s) have phosphoribosylamine-glycine ligase activity, phosphoribosylformylglycinamidine cyclo-ligase activity
AFUA_6G05080		ABC transporter, ortholog in *S. cerevisiae* involved in RNA regulation and maturation
AFUA_6G06870		casein kinase I homolog, ortholog(s) have magnesium ion binding, protein serine/threonine kinase activity
AFUA_6G07540		t-complex protein 1, epsilon subunit, ortholog(s) have chaperonin-containing T-complex localization
AFUA_6G07640		lysyl-tRNA synthetase,
AFUA_6G12630		leucyl-tRNA synthetase, putative tRNA charging
AFUA_6G12820	mpkB	MAP kinase MpkB, orthologs are regulating carbon utilization
AFUA_7G05660		translation elongation factor eEF-3
Transcriptional control		
AFUA_2G11780		C2H2 transcription factor (CreA) ortholog(s) have carbon catabolite repression
AFUA_2G13260	medA	transcriptional regulator of adherence, host cell interactions and virulence; required for wild-type levels of conidiation
AFUA_2G16230		C2H2 finger domain protein, putative transcription factor, orthologs have predicted regulation of gluconeogenesis and adherence
AFUA_2G17220		C2H2 transcription factor (AmdX), *A. nidulans* ortholog involved in regulation of acetamide catabolism; regulates amdS transcription
AFUA_3G08010	ace1	C2H2 transcription factor (Ace1), involved in cation homeostasis and in response to abiotic stress in *A. nidulans*
AFUA_3G10300		putative galactokinase ortholog in *S. cerevisiae* involved in conversion of alpha-D-galactose to galactose-1-phosphate, a key step in galactose catabolism
AFUA_3G10530		protein serine/threonine kinase (Ran1), orthologs have negative regulation of transcription/ development-meiosis
AFUA_3G11250	ace2	C2H2 transcription factor (Swi5), role in conidiophore development, pigment production, germination and virulence
AFUA_4G10220		homeobox transcription factor (RfeB), ortholog(s) have role in conidiophore development, conidium formation, hyphal growth, regulation nucleotide biosynthesis
AFUA_4G11480		C2H2 finger domain protein, *S. cerevisiae* ortholog is Zinc-regulated transcription facto
AFUA_4G12470	cpcA	bZIP transcriptional activator of the cross-pathway control system of amino acid biosynthesis upon starvation
AFUA_5G06190	steA	putative transcription factor involved in sexual development
AFUA_6G02110		MADS box transcription factor Mcm1, orthologs are involved in cell cycle
Energy		
AFUA_6G12930	acoA	aconitate hydratase, is essential for energy metabolism, TCA cycle and lysine biosynthesis [59]
Transmembrane transport GO:0055085		
AFUA_1G05020		putative sulfate transporter
AFUA_2G03860	zrfB	plasma membrane low affinity zinc ion, Low affinity plasma membrane zinc transporter; induced by zinc depletion
AFUA_2G14570		zinc/cadmium resistance protein, ortholog(s) have zinc ion transmembrane transporter activity, role in cellular zinc ion homeostasis
AFUA_3G12900	hasB	MFS transporter, orthologous member of the “has” secondary metabolite biosynthetic gene cluster of strain A1163. [67]
AFUA_4G14230		MFS transporter, putative transporter of small solutes
AFUA_4G14670		MFS quinate transporter, has domain(s) with predicted substrate-specific transmembrane transporter activity
AFUA_5G00980		MFS multidrug transporter, putative transporter of small solutes
AFUA_5G02700		MFS multidrug transporter, putative multidrug resistant protein
AFUA_7G01790	ssu1	MFS transporter, putative sulphite efflux pump
AFUA_7G05220		putative mitochondrial carrier protein
AFUA_7G05420		mitochondrial intermembrane space protein Mia40, putatively involved in intermembrane space import
180 min up-regulation		
DNA repair		
AFUA_2G02090	agt	O6-alkylguanine DNA alkyltransferase; role in DNA repair
AFUA_3G11610	nhp6	nucleosome binding protein, non-histone chromosomal protein ortholog in S. cerevisiae play role in elongation and DNA repair
Transcription factors		
AFUA_2G15680		transcription initiation factor iia small chain, ortholog(s) have TBP-class protein binding, activity involved in preinitiation complex assembly activity
AFUA_4G03960	farA	C6 transcription factor Ctf1A, role in beta oxidation of long-chain fatty acids
AFUA_7G06320		C6 transcription factor, has domain(s) with predicted DNA binding, sequence-specific DNA binding RNA polymerase II transcription factor activity
Protein binding, degradation, modification		
AFUA_5G05790		ubiquitin ligase subunit HrtA, ring finger protein rbxA, putative E3 ubiquitin-protein ligase; NeddH-associated protein
AFUA_6G09160		ubiquitin conjugating enzyme Ubc8, negatively regulates gluconeogenesis by mediating the ubiquitination of fructose-1,6-bisphosphatase [70]
AFUA_6G14210		ubiquitin conjugating enzyme UbcB, ortholog(s) have ubiquitin-protein ligase activity
AFUA_1G07470	atg8	autophagic death protein atg8, ribo involved in autophagy, determines the size of the autophagosome, induced upon starvation
Systemic interaction with the environment		
AFUA_4G00660		sensor histidine kinase/response regulator, putative stress sensor
Nitrilases/ cyanid hydratases		
AFUA_6G13450		nitrilase, ortholog(s) have cyanide hydratase activity, nitrilase activity and role in cyanide catabolic process
AFUA_2G17500		cyanide hydratase/nitrilase, ortholog(s) have cyanide hydratase activity, nitrilase activity and role in cyanide catabolic process
Oxidation-reduction process GO:0055114		
AFUA_2G10960	adh2	putative alcohol dehydrogenase Adh2p, in *S. cerevisiae* is responsible for catalyzing the initial step in the utilization of ethanol as a carbon source
AFUA_2G11250		aryl-alcohol dehydrogenase Aad14, unknown function
AFUA_2G15930		alcohol dehydrogenase, ortholog in *S. pombe* role in detoxifying alcohols and related compounds, protecting against environmental stresses
AFUA_4G12990	trr1	thioredoxin reductase Trr1/Trr2, orthologs play a role in detoxification
Iron homeostasis [12]		
AFUA_3G03640	mirB	MFS siderochrome iron transporter MirB,
AFUA_3G03440	mirD	MFS siderophore iron transporter, expression upregulated under low iron condition
AFUA_7G06060	sit1	siderochrome-iron transporter Sit1,
AFUA_1G17270	fre2	FRE family ferric-chelate reductase, metalloreductase involved in response to iron starvation; reductive iron assimilation, freB
AFUA_3G03410	sidH	enoyl-CoA hydratase/isomerase family protein, mevalonyl-CoA hydratase; is essential for (TAFC) biosynthesis
AFUA_3G03400	sidF	siderophore biosynthesis acetylase AceI, hydroxyornithine transacylase; involved in extracellular siderophore biosynthesis; essential for TAFC biosynthesis
AFUA_3G03420	sidD	nonribosomal peptide synthase SidD, fusarinine C non-ribosomal peptide synthetase (NRPS) involved in extracellular siderophore biosynthesisthesis
AFUA_3G03430	sitT	ABC multidrug transporter SitT, involved in siderophore excretion
AFUA_3G03440	mirD	MFS siderophore iron transporter,
AFUA_2G07680	sidA	L-ornithine N5-oxygenase SidA, first committed step in siderophore biosynthesis; TAFC and ferricrocin biosynthesis
Reductive iron assimilation		
AFUA_5G03790	fetC	ferrooxidoreductase Fet3,; reductive iron assimilation, FetC
AFUA_5G03800	ftrA	high-affinity iron transporter FtrA, reductive iron assimilation, FtrA
Gluconeogenesis		
AFUA_6G07720	acuF	phosphoenolpyruvate carboxykinase AcuF, essential for gluconeogenesis
AFUA_4G11310	fbp1	fructose-1,6-bisphosphatase Fbp1, essential for glucenogenesis
AFUA_6G09160		ubiquitin conjugating enzyme Ubc8, n negatively regulates gluconeogenesis by mediating the ubiquitination of fructose-1,6-bisphosphatase [70]

Adjusted categories and curated DEGs from FunCat and GO functional analysis that are refered to in the text. DEGs were manually investigated using the Aspergillus Genome Database, http://www.aspergillusgenome.org/ (ASPGD). The known description, putative function or ortholog function obtained are directly cited from ASPGD, from cited paper or description was obtained from link to *S. cerevisiae* genome project (http://www.yeastgenome.org/), from *Candida* Genome Database (http://www.candidagenome.org/), from *S. pombe* pombase (http://www.pombase.org/) [92], and from *Neurospora crassa* Sequencing Project Broad Institute of Harvard and MIT (http://www.broadinstitute.org/annotation/genome/neurospora/MultiHome.html)

### Changed expression after 30 min in blood primarily connected to up-regulated genes

The functional enrichment analysis revealed that after 30 min in blood (B30s condition) most significantly enriched categories correspond to up-regulated genes and fewer to down-regulated genes (Fig. 4 and Additional file 4: Table S3). The categories metabolism, and cell rescue, defense and virulence are enriched in both up- and down-regulated genes. The categories mostly enriched in up-regulation are transcription, protein fate, protein synthesis, and transport. The category enriched by down-regulated genes is cell cycle and DNA processing.

#### Metabolism

**Fig. 5:**
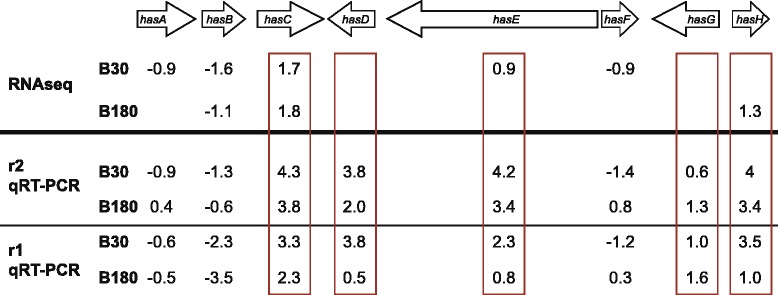
Regulation of the hexadehydroastechrome (HAS) cluster during blood incubation using RNAseq and qRT-PCR data. Genes from the HAS cluster of secondary metabolites showed different regulation pattern. All values are fold change (log2). The qRT-PCR data were obtained using experiment r1 and r2 in the same way as described in Additional file 3: Figure S1B. The red rectangles show up-regulated genes in blood. Empty cells in the RNAseq did not have any significant differential expression of the corresponding gene at this condition

The metabolism category includes primary metabolism as amino acid metabolism, lipid, fatty acid and isoprenoid metabolism, sulfur metabolism and also secondary metabolism. Up-regulated genes are involved in the metabolism of the amino acids arginine or histidine and to connections between amino acid metabolism and pyrimidines, purines and carbohydrates. As down-regulation for the category metabolism amino-acid metabolism especially, metabolism of the aspartate family and the pyruvate family are significantly enriched. The category lipid, fatty acid and isoprenoid metabolism bears genes that are up- and down-regulated. Two of the up-regulated genes from this category, AFUA_8G04080 (*badA*) and AFUA_8G04090 (*codA*), are involved in glycine betaine biosynthesis (Table 2). Glycine betaine was proposed to be used as an alternative nutrition pathway for amino acid biosynthesis upon nitrogen starvation in *A. fumigatus* [45]. Thus it is more suitable to add these genes into the category nitrogen, sulfur and selenium metabolism. From the down-regulated genes related to lipid, fatty acid and isoprenoid metabolism many genes were found to be involved in the ergosterol pathway, the main sterol of the fungal plasma membrane. The early down-regulation of ergosterol biosynthesis is remarkable since it is a main target for antifungal drugs like amphotericin B that is currently used for aspergillosis [46]. The category secondary metabolism is enriched in the condition B30 for up- and down-regulated genes. Only three from the 11 genes that are up-regulated can be directly related to secondary metabolism production and for two the secondary metabolite is known (Table 2 and Fig. 5, see below for further description). From the secondary metabolite category two genes that are down-regulated are linked to fumigaclavine C or fumarylalanine production called *rps1* and *sidE*, respectively [47, 48]. Fumarylalanine was described as immunomodulatory drug and indeed *sidE* is up-regulated in murine lung infection [26, 48]. Rps1 was shown to have an influence on oxidative stress and virulence in the insect infection model *Galleria mellonella* [49]. Therefore it is likely that these secondary metabolite products are needed for infection but dispensable for the survival in blood.

#### Transcription

In the category transcription the putative and known function of the corresponding genes that are up-regulated is in RNA synthesis, processing, modification and in ribosomal RNA synthesis, processing, and modification. The FunCat subterm ‘transcriptional control’ contains 23 genes that are up-regulated in B30s. Most genes of this category have a more general role in transcription (Table 2). Only few genes are known that show an obvious regulatory role in specific pathways. This is AFUA_5G06190 (SteA), whose orthologous gene in *A. nidulans* encodes a transcription factor that is involved in sexual development [50], AFUA_5G05610 encoding a putative cell cycle control protein and AFUA_1G12940 [51] for a MAP kinase that regulates transcriptional stress response and plays a role in nitrogen starvation [52]. A number of transcription factor-encoding genes are down-regulated. These transcription factors are primarily involved in DNA processing, development or metabolism.

#### Cellular transport

A number of genes which are up-regulated at B30s are involved in a variety of transport processes (Table 2). Some genes encode predicted electron transport and one gene is for a putative toxin transporter. Only two genes for putative amino acid transporters show differential transcription. AFUA_7G04290 (GapA), a general amino permease transporting most amino acids, and AFUA_8G02200 (for proline permease) are up-regulated at the less stringent condition B30ls.

#### Cell rescue, defense and virulence

The categories cell rescue, defense and virulence are enriched in up- and down-regulated genes at B30s. The subterm stress response is related to up-regulated genes. These include genes predicted to be involved in DNA repair and in chaperone and protein folding activity (Table 2). To the latter category belong AFUA_2G02320 (Hsp70 chaperone BiP) AFUA_4G03650 (ribosome associated DnaJ chaperone Zuotin), AFUA_8G03930 (HscA), AFUA_2G16290 (DnaJ domain protein Mas5) and AFUA_4G12360 (prefoldin subunit 1). SteA and SakA already mentioned in the category transcription can also be related to stress response and additionally AFUA_5G11230 (RasA), is a GTPase that is involved in asexual development and cell wall integrity [53]. Also predicted detoxification proteins like the thioredoxin reductase AFUA_4G12990, two putative transporters AFUA_4G00930 and AFUA_1G12620 and the predicted hydrolase AFUA_2G13080, are up-regulated in the B30s data set. The down-regulated genes from this category belong to the subcategory resistance protein and are putative transporters like AFUA_8G02050 (putative polysaccharide transporter), AFUA_1G03200 (MFS transporter), AFUA_1G10370 (MFS multidrug transporter), AFUA_1G10390 (ABC multidrug transporter), AFUA_1G13800 (MFS multidrug transporter), AFUA_6G02220 (MFS toxin efflux pump), AFUA_5G02700 (MFS multidrug transporter), and AFUA_7G01790 (MFS transporter). Also genes putatively involved in protein folding and heat shock are down-regulated (Table 2). HscA and thioredoxin reductase were also confirmed by qRT-PCR analysis (Additional file 3: Figure S1C).

#### Cell cycle and DNA processing

Cell cycle and DNA processing is a category that is only significantly enriched for down-regulated genes at 30 min. These are predicted genes mainly involved in DNA processing, in DNA restriction or modification, and in DNA recombination and DNA repair. Several histones belong to the list of down-regulation in B30s (Table 2). Also some transcription factors, kinases and genes putatively involved in cytoskeleton formation like AFUA_2G14990 (tubulin), AFUA_7G01400 (kinesin family protein BimC), AFUA_3G06280 (Rho GTPase activator Rgd1), AFUA_1G13750 (C2H2 transcription factor Rpn4), AFUA_2G17220 (C2H2 transcription factor AmdX), and AFUA_3G11250 (C2H2 transcription factor Swi5) are in this list. Rpn4 down-regulation was also confirmed at qRT-PCR (Additional file 3: Figure S1C).

### Genes up-regulated after 30 min but down-regulated after 180 min in blood

These categories of differentially expressed genes show an opposing regulation pattern after short or longer blood exposure. They are first up-regulated in B30s and later down-regulated in B180s. Further investigations of the DEGs included in these categories showed a subset of them appearing in the 30 min and the 180 min list simultaneously. However, most of the other DEGs that appear in one list only show the same regulation pattern but fall below the threshold of 30 min or 180 min.

So the majority of the DEGs described here have a similar regulation pattern, although not all are represented in both conditions (Additional file 4: Table S3).

#### Protein synthesis and fate

A large number of DEGs belong to the category ‘protein synthesis’ and have the early up- and late down-regulation pattern. They belong to the subcategories ribosome biogenesis, translation, translational control and translational initiation. Synthesis of ribosomes is a very energy-consuming mechanism [54]. The early up-regulation reflects the fungus is investing energy for protein synthesis and hence metabolic changes whereas the shut off of metabolic activity reflects down-regulation and energy saving.

Protein fate with the subterms protein modification and protein/ peptide degradation is enriched for up-regulated genes in B30s. Genes that are predicted to belong to this category are AFUA_1G04040 (UbiA) and AFUA_5G06060 (SkpA) which are part of SCF ubiquitin ligase complex [55]. This complex is necessary to connect ubiquitin to the substrate that is bound to the F-box protein and therefore directs it to protein degradation at the 26S proteasome [56]. With the ubiquitination system the fungus has a tool to change the stability, localization or activity of a protein. The protein degradation also involves the following other proteins: AFUA_5G04000 (proteasome maturation and ribosomal biogenesis), AFUA_4G10070 (class I α-mannosidase, involved in ER protein degradation) and various peptidases with unknown function. Also genes involved in glycosylation and phosphorylation are modifying proteins for localization and activity and show the early up- and late down-regulation pattern (Table 2).

### Genes down-regulated after 180 min in blood

As described in the previous pattern at 180 min nearly all significantly enriched categories correspond to down-regulated genes. The categories metabolism, protein biosynthesis, protein fate, transport and energy are all related to down-regulated genes.

#### Metabolism

A number of genes which are down-regulated at B180s enlisted to subcategories that are involved in the biosynthesis or degradation of amino acids. This includes not only genes with direct involvement of amino acid biosynthesis and degradation but also genes that are predicted to be involved in purine metabolism, nitrogen and carbon utilization, polyamines synthesis and secondary metabolites. Assimilation of ammonia, metabolism of the glutamate group is a category belonging to down-regulated genes. Most of the genes in this list are connected to TCA cycle, and transamination. But also genes are down-regulated that directly relate to glutamate degradation as nitrogen source like the predicted gene AFUA_2G06000 (NAD+ dependent glutamate dehydrogenase) as well as for the glutamate degradation via the GABA shunt pathway the predicted genes AFUA_6G13490 (glutamate decarboxylase) and AFUA_5G06680 (4-aminobutyrate transaminase GatA). The categories metabolism of the cysteine aromatic group has several genes for aromatic amino acid production that are down-regulated. These are specifically the genes for aromatic amino acid biosynthesis such as AFUA_5G13130 (chorismate mutase) and especially tryptophan production, a highly energy consuming pathway [57]: AFUA_1G13090 (multifunctional anthranilate synthase), AFUA_4G11980 (anthranilate phosphoribosyltransferase), AFUA_2G13250 (bifunctional tryptophan synthase TrpB). In the category ‘C-compound and carbohydrate metabolism’ several of the down-regulated genes, are predicted to be involved in sugar, glucoside, polyol and carboxylate anabolism.

#### Energy

The subcategory ‘tricarboxylic acid cycle (TCA), electron transport chain’ contains 43 down-regulated genes (Additional file 4: Table S3). They are predicted to be related to aerobic respiration, TCA cycle, electron transport chain, and metabolism of energy reserves. Several genes involved in electron transport chain that are down-regulated are directly involved in the process as: AFUA_1G02070 (putative mitochondrial Cytochrome C1), AFUA_4G03120 (putative mitochondrial cytochrome b2), AFUA_1G03510 (putative mitochondrial ATP synthase), AFUA_3G10110 (putative electron transfer flavoprotein-ubiquinone), and several NADH-ubiquinone dehydrogenase. AFUA_3G07810 (predicted succinate dehydrogenase, *sdh1*) and AFUA_5G10370 (predicted iron-sulfur protein subunit of succinate, *sdh2*) are additionally to electron transport chain involved in the TCA cycle. In contrast to *S. cerevisiae* which can ferment, the TCA cycle is necessary for gaining energy in *A. fumigatus* [58]. Several genes which are involved in the TCA cycle are down-regulated: AFUA_1G06810 (putative aconitate hydratase, mitochondrial), AFUA_3G08660 (putative isocitrate dehydrogenase Idp1), AFUA_5G04230 (citrate synthase Cit1) and AFUA_6G12930 (AcoA), an aconitate hydratase which is essential for energy metabolism in *A. fumigatus* (Table 2). Since AcoA is essential for growth and the deletion mutant of the corresponding gene in not viable, the down-regulation at 180 min suggests that *A. fumigatus* is virtually not generating energy [59]. AcoA is not only responsible for citrate to isocitrate conversion in the TCA cycle but also for homocitrate to homoisocitrate conversion during lysine biosynthesis. So it is consistent that two genes for the α-aminoadipate pathway for lysine biosynthesis are also down-regulated: AFUA_6G07390 (LysB) a putative homoisocitrate dehydrogenase and AFUA_3G11710 a saccharopine dehydrogenase. This is an interesting aspect in respect to secondary metabolism production, since lysine is necessary for β-lactam antibiotics production [60].

In the less stringent condition B180ls the category glycolysis is significantly enriched for down-regulated genes, so again energy production is down-regulated. One example AFUA_2G09790 (glucose-6-phosphate isomerase) was also confirmed in qRT-PCR (Additional file 3: Figure S1C) The category metabolism for energy reserves include the genes predicted for glycogen biosynthesis AFUA_5G02480 (glycogen synthase Gsy1), AFUA_2G13850 (protein phosphatase regulatory subunit Gac1), and trehalose biogenesis AFUA_2G04010 (alpha,alpha-trehalose-phosphate synthase). Also AFUA_3G11830 (phosphoglucomutase PgmA) is down-regulated at B180s which is involved in glycolysis as well as glycogen and trehalose biogenesis [61].

#### Mitotic cell cycle and cell cycle control

The category mitotic cell cycle and cell cycle control is highly enriched with 36 down-regulated genes (Additional file 4: Table S3). In this category are predicted kinases, phosphatases, cytoskeletal proteins, and histone related proteins already described in other paragraphs.

#### Transcription

Transcriptional control shows a number of down-regulated genes (Table 2). In this category is AFUA_4G12470 (CpcA) a transcription factor that is required for the cross-pathway control, a mechanism that provides amino acid supply during amino acid starvation by increasing the synthesis of biosynthetic genes. CpcA is partially required for virulence [15] and its transcription pattern could be confirmed by qRT-PCR (Additional file 3: Figure S1C). Further genes from this category are AFUA_2G13260 (MedA), a developmental modifier that is also required for adherence and furthermore plays a role in virulence [62], AFUA_5G06190 (SteA) a transcription factor predicted to be involved in development and sexual reproduction [50], AFUA_2G17220 C2H2 transcription factor AmdX putatively required for growth and nitrogen starvation, AFUA_3G10300 a putative galactokinase involved in stress response, and AFUA_2G11780 C2H2 transcription factor with homology to the carbon catabolite repressor CreA [63].

#### Transmembrane transporter

The GO category ‘transmembrane transporter’ is exhibiting a down-regulation pattern. The function of the transporters is in most cases yet unknown, but they are often predicted to be multidrug transporter. Any involvement in up-take mechanisms related to metabolism is with the current state of knowledge yet elusive.

#### ATP binding

This GO category has a large number of genes that are involved in a variety of biological pathways. The fungus seems to reduce ATP binding and consumption which is consistent with the finding of the same pattern of down regulated genes for ribosomal and protein biosynthetic pathways which are also highly energy consuming.

#### Disease, virulence and defense

The less stringent list of down-regulation for B180ls significantly enriches the category ‘disease, virulence and defense’. The genes belonging to this category are transporters, but also AFUA_3G03350 (SidE), which is already down-regulated at 30 min and is involved in fumarylalanine production [48].

### Up-regulated genes after 180 min in blood

Very few categories are enriched that correspond to up-regulated genes in the B180s condition.

#### Transcription

In the category ‘transcription’, the 11 genes up-regulated are involved in DNA repair, three genes encode predicted transcription factors of yet unknown functions.

#### Protein binding, degradation, modification

AFUA_5G05790 is up-regulated and encodes the RbxA homolog for the RING protein which is the catalytic subunit of E3 ubiquitin ligases for protein modification. Modification by ubiquitin changes protein stability, function or localization. Further genes involved in ubiquitin modifications which often increase protein instability are AFUA_6G09160 (ubiquitin conjugating enzyme Ubc8) and AFUA_6G14210 (ubiquitin conjugating enzyme UbcB). Also AFUA_1G07470 (autophagic death protein Atg8) is up-regulated in B180s. Autophagy is a possibility for the fungus to deal with nutrient starvation like nitrogen and iron shortage by recycling its own proteins and is an alternative to proteasomal degradation via the ubiquitin system [64]. Atg8 is required for autophagosome formation and the mRNA is up-regulated during starvation conditions in *S. cerevisiae* [65, 66]. It is possible that protein stability, modification and function are required to be active at 180 min or to be maintain an alert status to readily react on environmental changes.

#### Detoxification and stress

The enriched category systemic interaction with the environment reveals that two predicted nitrilases or cyanid hydratases and AFUA_4G00660, a sensor histidine kinase/response regulator predicted to be involved in osmotic stress, are up-regulated at B180s. The GO term oxidation-reduction process reveals that some genes that are predicted to be involved in oxidative stress are up-regulated at B180s like three alcohol hydrogenases AFUA_2G10960 (alcohol dehydrogenase Adh2), AFUA_2G11250 (aryl-alcohol dehydrogenase Aad14), AFUA_2G15930 (alcohol dehydrogenase, zinc-containing) and the putative thioredoxin reductase encoding gene AFUA_4G12990 (Aspf29, an allergen and putative thioredoxin TrxA).

#### Iron starvation

Under less stringent evaluation, siderophore-iron transport is significantly enriched for up-regulated genes during the late stage. More detailed investigation revealed that several genes of iron homeostasis are up-regulated at B180 in RNA-Seq data as well as in qRT-PCR results (Additional file 3: Figure S1A) [12]: AFUA_3G03640 (MFS siderochrome iron transporter MirB), AFUA_3G03440 (MFS siderophore iron transporter), AFUA_7G06060 (siderochrome-iron transporter Sit1), AFUA_1G17270 (FRE family ferric-chelate reductase), AFUA_3G03410 (enoyl-CoA hydratase/isomerase family protein SidH), AFUA_3G03400 (siderophore biosynthesis acetylase AceI), AFUA_3G03420 (nonribosomal peptide synthase SidD), AFUA_3G03430 (ABC multidrug transporter SitT), AFUA_3G03440 (MFS siderophore iron transporter), and AFUA_2G07680 (L-ornithine N5-oxygenase SidA) are up-regulated at B180ls. The transporter for reductive iron assimilation AFUA_5G03790 (ferrooxidoreductase FetC) and AFUA_5G03800 (high-affinity iron transporter FtrA) are down-regulated at B30s (Additional file 3: Figure S1A). The number of genes shows that iron starvation occurs in blood and that the fungus is forced to specifically up-regulate genes for siderophore biosynthesis. This is in consistency with the known iron starvation conditions during infection in pulmonary aspergillosis [67]. Also it was already described that *A. fumigatus* obtains iron from transferrin in human serum by secretion of siderophore [68]. Our study confirms on transcriptional level that the fungus produces siderophores in blood. The supply of iron seems to be more important than to down-regulate the siderophore biogenesis in order to save energy. This is most probably because it is needed for several important processes like electron transport, amino acid metabolism, DNA biosynthesis and must be stored to have a rapid supply when growth is reinitiated [12].

#### Gluconeogenesis

Gluconeogenesis is needed when glucose is no longer available in the medium to produce hexose sugars. Glucose is present in blood in a lower level (<1.0 g/l) than in minimal medium (10 g/l) [69]. The predicted genes that are essential for the enriched category gluconeogenesis are down-regulated in the early stage at B30s: AFUA_6G07720 (phosphoenolpyruvate carboxykinase AcuF), AFUA_4G11310 (fructose-1,6-bisphosphatase Fbp1) and the negative regulator AFUA_6G09160 (ubiquitin conjugating enzyme Ubc8) which is involved in numerous cellular processes. This suggests that glucose levels might be initially still sufficient for the fungus after the transfer to blood [70]. However, mRNAs levels encoding these enzymes appear to increase again at B180 min. For Fbp1 belonging to the B180s list of up-regulated genes, whereas *acuF* regained pre-expression levels and does therefore not appear in the B180s list. Transcript levels of the negative regulator Ubc8 in contrast do not recover. This might be a secondary effect of the experimental setup. *In vitro* glucose might be exhausted at 180 min and the fungus prepares for alternative carbon source metabolism and therefore shows an earlier up-regulation of gluconeogenesis genes than the minimal medium control. In the bloodstream there would be enough glucose available, due to continuous supply. So it can be assumed that the activation of gluconeogenesis is not an active process occurring in the host bloodstream.

### The hexadehydroastechrome secondary metabolite cluster is partially up-regulated in blood

Two of the three secondary metabolite genes up-regulated in B30s belong to a cluster that is able to synthesize hexadehydroastechrome (HAS cluster) which might be involved in fungal virulence [67]. We analyzed the expression levels in our experimental setup for the eight genes belonging to this cluster and found that six genes of this cluster are significantly differentially expressed (Fig. 5). Three genes are down-regulated (*hasA* and *hasF* at B30s and *hasB* at B30s and B180s), two are up-regulated at B30s exclusively (*hasC* and *hasE*), and one is up-regulated at B180s (*hasH*). Further analysis of the RPKM-corrected expression levels revealed that *hasD* and *hasE* show higher expression levels in B30s or B180s, respectively, but fall below the threshold. qRT-PCR data showed similar up-regulation and down-regulation pattern of the genes and the results are summarized in Additional file 3: Figure S1B and Fig. 5.

The HAS-cluster is a secondary metabolite cluster that is silent under laboratory culture conditions [71, 72]. Overexpression of *hasA*, a putative transcription factor, leads to expression of all members of the cluster and consequently to synthesis of HAS. Additionally, *hasA* overexpression leads to increased virulence of *A. fumigatus* in the mouse model albeit HAS, the end-product of this cluster, could not be detected [67]. HAS synthesis is based on *L*-tryptophan and *L*-alanine, so therefore is a highly energy consuming pathway (see also tryptophan biosynthesis at 180 min). It contains Fe(III) and *in vitro* iron depletion abolished the production of HAS. Terezine D,7 an intermediate of the HAS production with unknown function, was detected in murine infections [67]. The regulation data from this study reveal that most likely the whole cluster is not up-regulated in blood but instead the regulatory proteins like transcription factors and transporters (HasA, HasB, and HasF) are down-regulated. Hence, the up-regulation of *hasC, hasE*, and *hasH* is executed by an unknown transcription factor and independent of *hasA* regulation. They lead to a different product than HAS, which could be Tresine D,7 or an unidentified product. These results are in line with the results from [67] and are also supported by the transcriptome analysis from the mouse model that showed exactly the same regulation pattern for the cluster as our *in vitro* blood results [26].

### Summarizing the fungal gene expression response after 30 min in blood

Our data show that after 30 min in blood the fungus specifically controls cellular transport, protein synthesis, and metabolism. Metabolism reflects the response to the environment how the fungus is obtaining nitrogen, carbon, and other nutritional needs. The up-regulation of genes belonging to amino acid metabolism and nucleoside, nucleotide or nucleobase metabolism can be related to nitrogen metabolism and as well to transcription in general. No obvious changes in genes for pathways for energy metabolism can be detected. Only the genes involved in glycine betaine biosynthesis might be related to starvation conditions [45], but the known factors for other starvation conditions are not up-regulated. Hence the genes related to the target of rapamycin (tor) pathway, GABA shunt, *areA* and the genes from transcriptomic analysis of nitrogen starvation are not up-regulated [26, 73, 74]. Also key genes for gluconeogenesis (*acuF*), amino acid starvation (*cpcA*) hypoxia response (*srbA*) and iron starvation are not increased at the early blood incubation condition [12, 14, 15, 75].

It is thought that *A. fumigatus* feeds mainly on amino acids during infection. This is based on the observation that uptake of isoleucine, valine and methionine for the methylcitrate cycle is a prerequisite for infection [76]. Interestingly two amino acid transporters are up-regulated at the early phase in blood with one being a general permease and no transporter is up-regulated in the late phase. It is possible that the amino acid uptake at the 30 min time point occurs unspecific. The fungus negotiates the supply of amino acids and decides what is needed. A specific uptake is then no longer needed at the later stage. Some amino acid biosynthesis genes are up-regulated in the early phase, however more likely related to purine and pyrimidine biosynthesis, which again might account for the adaption process and need of protein production. The elevated category transport reflects that the fungus does take up compounds for metabolism and energy production. As mentioned above glucose is present in blood. Glucose has a function as catabolite repressor [77], so it can be expected that the fungus does not change the energy metabolism compared to minimal medium in the early phase of blood incubation and indeed in the category energy no directly related genes were up-regulated at this early time point. Categories involved in growth like cell cycle, DNA synthesis, ergosterol biosynthesis and cell wall synthesis are connected to mostly down-regulated genes. When summarizing all categories enriched it becomes obvious that the fungus is not in a growth but in an adaption phase at 30 min, shutting down and inducing different metabolic pathways in order to synthesize new proteins to deal with the new condition. Hence also genes for secondary metabolism and cell rescue defense and virulence show both, up-and down-regulation. So the stress response and defense strategies are adapting during this condition.

### Summarizing the fungal gene expression response after 180 min in blood

Most of the categories enriched in the late stage show down-regulation. This reflects that the fungus is massively slowing down metabolism, saving energy, and is in a no growth phase. This is a specific activity and not due to an exhaust of nutrition, shown by the down-regulation of known transcription factors that should be induced under starvation conditions like *cpcA*, *areA*, and also genes for amino acid degradation and biosynthesis. Categories for transport, transcription factors and ATP-binding are down-regulated presumably to save energy. Genes like the essential aconitase hydratase *acoA* [59] or ribosome synthesis reflects that energy saving is the main focus at this condition. Also genes for tryptophan biosynthesis, a very cost intensive amino acid production are down-regulated. The shut-down of most of the energy consuming pathways might be interpreted as a defense strategy to hide until environmental changes enable the fungus to grow. This might explain why the fungus is not found at high levels in blood during invasive aspergillosis but instead it can only actively grow by colonizing tissue to get all the nutrients necessary for active growth. The assumption that the fungus is literally resting in the late phase, corresponds to our observation that after 24 h in blood the mycelium did not show any growth but transition to new medium reinitiated growth again (data not shown). Our attempts to quantify metabolic activity of the fungus under this experimental condition failed due to a significant auto-fluorescence of blood.

The exception of the energy saving mode is the up-regulation of mechanisms putatively needed for active protection and survival in blood. Hence expression is more important for survival than energy conservation aspects. The mechanisms are some genes from protein degradation and autophagy, genes from detoxification processes by nitrilases but also for coping with starvation conditions for iron. Gluconeogenesis is most probably not an active process for blood survival but an artifact due to the experimental setup as described earlier. Functional evidence about the high number of DEGs coding for hypothetical proteins in the condition B180s would probably reveal more survival strategies of the fungus in the blood environment that are still unknown.

The iron homeostasis, corresponds to the known iron starvation conditions during infection in pulmonary aspergillosis [67]. The second active protection mechanism is related to detoxification, like thioredoxin, alcohol dehydogenase and cyanid hydratase [78–80]. All these proteins have orthologs that play a role in stress response but they have not been studied in detail in *A. fumigatus*. Also the partial up-regulation of the secondary metabolite cluster HAS, which starts already at 30 min, but the last member is up-regulated at 180 min might play a role in actively ensuring survival in blood. This cluster is especially interesting because it is not expressed under different laboratory conditions and the same partial regulation of the genes in this cluster was also observed in the mouse model transcriptome [26]. In a phase in which most of the energy consuming pathways are down-regulated it is not likely that the up-regulation of the HAS genes utilizing, tryptophan is unspecific. To verify that no other known secondary metabolite cluster was up-regulated we compared our data with the identified clusters that are affected by the methyltransferase LaeA. The transcriptome data, did not show much correlation to our results and also no LaeA independent cluster was up-regulated [38]. So we conclude that these secondary metabolite clusters are not needed to survive in or adapt to blood, hence these clusters might be needed at a later stage of infection. However, it is possible that unidentified genes participate on the production of unknown secondary metabolites that are needed for protection of the fungus in blood. The high number of up-regulated unknown genes might contribute for this.

### Comparison to other transcriptome assays

We also compared transcriptome analysis from other setups in *Aspergillus fumigatus.* The percentage of differentially expressed genes in these analysis ranges between 2 % of the total genes for dendritic cell exposure to 23 % for voriconazol exposure [33, 40]. The 6–8 % of DEGs from our study is located in the lower range compared to the other analysis. Again, little correlation is seen between known virulence factors and our blood incubation results. Besides the partial HAS cluster a second cluster showed a similar regulation pattern in the mouse model transcriptome [26]. The second half of cluster 33 showed similar up-regulation of 5 out of the 14 genes. The genes regulated are involved in siderophore metabolism and TAFC biosynthesis. A consistency with iron starvation conditions was already mentioned above. Other comparisons using transcriptome data obtained by incubation with dendritic cells, neutrophils, upon nitrogen starvation, alkaline stress, oxidative stress did not show any strong similarities [26, 30, 33, 35]. We conclude that the dissemination process during aspergillosis leads to a very different reaction compared to the other stages of infection. The fungus seems not to proliferate and to take up any nutrients at all after a short adaption phase. Since massive proliferation is seen in the later dissemination state when the fungus colonizes other organs, it must be able to obtain enough nutrition for growth from the organs [8]. This is probably due to necrotic abilities on tissue, by secreting hydrolytic enzymes that are rapidly released upon contact with the tissue [81–83].

For *Candida albicans* it is described that during *in vitro* blood incubation it can be observed that the fungus is ready to leave the harsh environment of blood [41]. This can be seen by the transition from yeast form to hyphae. Hyphal specific genes, hydrolases and adhesion factors are up-regulated in late phases of blood incubation so that the fungus gains the ability to penetrate the tissue. A similar preparation of *A. fumigatus* to leave the blood is not seen in the transcriptome during blood incubation. But since no morphological different infectious state is described for *A. fumigatus* this seems to be impossible to detect. Making iron available through siderophore production might reflect that the fungus is preparing for an escape from blood and hence a re-initiation of growth. Differences to the *C. albicans* transcriptome in blood are also seen with other mechanisms. Iron deficiency for example is not a problem, since *C. albicans* can utilize haem from blood [84]. In the transcriptome the up-regulation of the glyoxylate cycle is not seen in *A. fumigatus.* Also less genes involved in antioxidative response are regulated. Hence for example neither SOD, nor catalase are up-regulated. One from the five catalase genes is even down-regulated at 30 min in *A. fumigatus*. A similar behaviour of *C. albicans* to *A. fumigatus* is seen when the yeast is incubated with neutrophils, where it underwent growth arrest and nutrient starvation [85].

### Conclusions

Invasive aspergillosis, an infection caused in most cases by *Aspergillus fumigatus,* starts in immunocompromised patients in the lung by inhalation. From there fungal hyphae can grow through the epithelial tissue and disseminate via the bloodstream to colonize other organs. Presumably the fungus only stays in short time periods in the blood since outgrowth from blood is difficult to achieve and is less efficient than in Candida. Little is known about the dissemination phase during invasive aspergillosis. It was suggested that hyphal fragments detach and spread to infect other organs [86]. From our transcriptome data we assume that the situation may be more complex. The fungus appears to sense the new environment and adapts within hours to it by shutting down all unnecessary pathways in order to hide as long as possible in the blood and to escape as soon as possible just keeping live saving mechanisms running. Moreover, the possibility of hematogenous dissemination by a Trojan horse mechanism, i.e. assisted by phagocytes of the host immune system, has to be taken into account [87], and it might well be possible that both dissemination processes occur simultaneously.

Transcriptome analysis can only supply limited information on gene expression levels. So further studies on the level of protein amounts are necessary to better understand the cellular status of *A. fumigatus* in blood. Also functional characterization of some of the large number of genes that are currently annotated with unknown function might shed light on the dissemination process during invasive aspergillosis.

### Methods

#### Strain, media, and preculture

The *Aspergillus fumigatus* ATCC46645 strain was used in this study [88]. The minimal medium used consists of 1 % glucose, 2 % salt Solution (26 g/l KCl, 26 g/l MgSO_4_, 76 g/l KH_2_PO_4_, 5 % (v/v) trace elements) pH 7.0 plus 5 mM NH_4_-tartrate. The blood was withdrawn from one female donor using heparinized syringes and was pooled and immediately used. Mycelium of the *A. fumigatus* strain was grown 16 h in minimal medium, harvested, and pooled before transfer to the experimental culture conditions.

#### *In vitro* blood assay

For each biological control the mycelium was split in approximately 2 ml into reaction tubes. One tube without new medium was harvested and the mycelium was shock frozen in liquid nitrogen yielding the pre value. Concurrently 10 ml of either medium or blood was added to the rest of the tubes, they were several times inverted and incubated on a rotator at 37 °C for 15, 30, 60, 90, 180 min followed by harvesting and shock freezing in liquid nitrogen.

### Ethics statement

The local ethics committee (Ethikkommission der Universitätsmedizin Göttingen, Von-Siebold-Str. 3, D-37075 Göttingen, Germany under the chair of Prof. Jürgen Brockmöller) expressed no ethical or legal concerns about the procedures as used in the present investigation and approved the procedures using human blood samples.

### RNA isolation and microarray

*A. fumigatus* mycelia was disrupted by grinding with liquid nitrogen, and total RNA was extracted with RNeasy plant (Qiagen, Germany) following the instructions by the manufacturer.

### RT PCR and quantitative PCR

DNase digestion and subsequent cDNA synthesis was carried out in duplicates for each sample using 0.8 μg of RNA with the QuantiTect Reverse Transcription Kit (Qiagen). Amplification was performed in a Light Cycler 2.0 (Roche) with the RealMasterMix SYBR ROX (5Prime) using 1 μl of a 1/10 dilution of cDNA and *A. fumigatus* primers (Additional file 5: Table S4). Amplification conditions were as follows: 50 cycles of 95 °C 18 s, 63 °C 45 s and an adjacent melting step (63–95 °C). The amount of gene of interest relative to akuA and *h2A* mRNA was quantified using the delta delta CT method with efficiency [89]. All qRT-PCR experiments were performed at least in duplicates [56].

### cDNA library construction and sequencing

For sequencing, a new RNA preparation was carried out using the same deep frozen and ground mycelium and the same method as for quantitative RNA. The preparation of cDNA libraries were carried out by GATC Biotech with two different methods. For the first replicate (r1) the time point pre, B30 and B180 the SMART protocol was used and for r1 M30 and M180 and all r2 time points the TrueSeq protocol was used. For the SMART protocol, reverse transcription was carried out with Oligo d(T) primers followed by second strand synthesis, coligation, nebulization and adapter ligation. The three tagged cDNA libraries were pooled and paired end sequencing (Read length 100 bp) was performed on Illumina HiSeq 2000 by GATC Biotech (Konstanz, Germany). For TrueSeq, PolyA RNA was isolated, fragmented and random primed first strand cDNAs synthesis was carried out. Afterwards second strand synthesis was performed, adapters were ligated and the 7 tagged-libraries were pooled for sequencing. Paired end sequencing (Read length 100 bp) was carried out on Illumina HiSeq 2000 by GATC Biotech (Konstanz, Germany).

### Mapping, batch correction and expression quantification

The sequencing reads were mapped to the *A. fumigatus* reference genome (strain Af293, version 14) which was downloaded from EnsemblFungi website [40] using the TopHat software [41]. In order to obtain the number of reads mapping to annotated genes (expression quantification), htseq-count tool was used (Anders S.: HTSeq: Analysing high-throughput sequencing data with Python. http://www-huber.embl.de/users/anders/HTSeq/doc/overview.html). Figure 1b show the total number of reads, the number and percentage of reads mapping to the reference genome (uniquely or to more than one position) and the number of reads allocated to annotated genes.

**Fig. 6:**
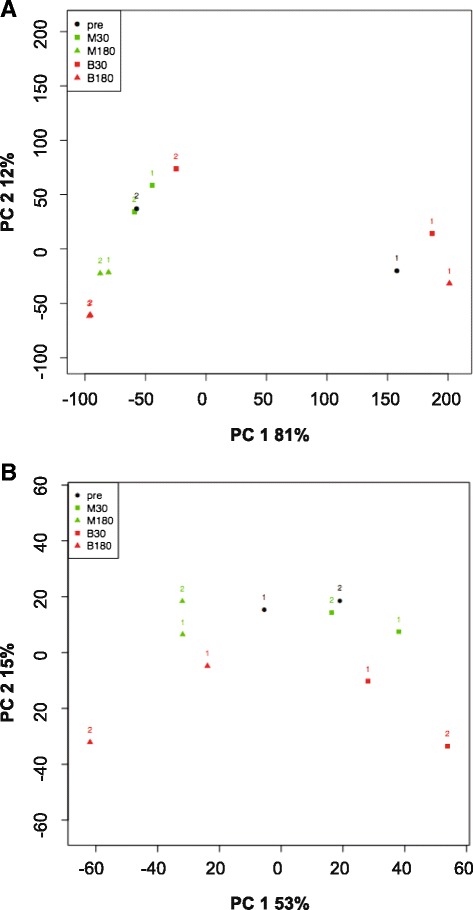
Principal component analysis (PCA). **a**: PCA on log_2_(RPKM + 1) values. **b**: PCA computed from data after ASCA correction of batch effect

The samples were not sequenced at the same time but in two different batches. A first exploratory Principal Component Analysis (PCA) revealed an important batch effect given that the first principal component separated the two batches and explained 81 % of the total variability (Fig. 6a). This batch effect caused that the correlation between replicates within the same experimental condition was sometimes lower than the correlation between conditions. We used ASCA method [90] on log_2_(RPKM + 1) in order to remove this batch effect from the data. ASCA first decomposes the data matrix into several submatrices, one per effect to be studied plus the residual one, which collects the part of the data than cannot be explained by any of the effects included in the analysis. Then, ASCA applies a PCA to each one of the submatrices. We included only the “batch” effect in the ASCA model and we subtracted from the data the part being explained by the batch effect. By doing this, those genes contributing most to the batch effect, are more corrected. Since not all the genes and samples are corrected in the same way, the resulting data have as a drawback that the expression for a given gene is not comparable among samples. We rescaled the data by applying quantile normalization to solve this problem and added a constant value to these quantile-normalized data to avoid negative expression values.

Figure 6b shows the PCA plot for the corrected data where no batch effect is observed. In this PCA, both conditions and replicates are properly clustered (with a correlation between replicates that varies from 0,95 to 0,97 and that is higher than the correlation between experimental conditions). Now, the first component explains 53 % of the variability in the data and it separates incubation time: the later time points in the left-hand side and earlier time points in the right-hand side. The second component separates blood from minimal medium samples.

The NOISeq method [42] was applied on the batch-corrected data to compute differential expression. Genes were declared as differentially expressed when the probability of differential expression was higher than 0.8. Additional file 2: Table S1 shows the differentially expressed genes (DEGs) for each comparison.

### Functional enrichment

Up-regulated or down-regulated genes from B30s, B30ls, B180s and B180ls as listed in Additional file 1: Table S2 were uploaded on the FungiFun platform with a *p*-value cutoff of 0.05 and analyzed for GO and FunCat functional enrichment [43].

### Availability of supporting data

The data discussed in this publication have been submitted to GEO/NCBI (accesion number GSE70227).

(http://www.ncbi.nlm.nih.gov/geo/query/acc.cgi?acc=GSE70227)

## Additional files

Additional file 1: Table S2. Sheet A: List of differential expressed genes in blood after 30 min and after 180 min in stringent (B30s/B180s) and less stringent (B30ls/B180ls) conditions. Sheet B and C: Genes included and excluded from the B30 (B) and B180 (C) lists were obtained by following method. Stringent (s) stands for no or opposite differential expression in the corresponding minimal medium condition. The list of genes with differential expression under less stringent conditions (ls) was obtained by adding those genes to the stringent condition lists that were differentially expressed to lower or higher extend than the blood values. A gene was included when the difference of the M value was more than 0.8 for positive values or less than −0.8 for negative values in the comparison M30/M180-pre to B30/B180-pre. M represents the log2-ratio or fold change for positive (up-regulated) and negative (down-regulated) genes. D is the absolute value of the difference. Prob. is the probability of differential expression obtained by NOISeq method. (XLS 1261 kb) Additional file 2: Table S1. List of differential expressed genes at different comparisons. Positive values show up-regulation within the first condition compared to the second condition. Down-regulation accordingly is represented by negative values. The value represents the log2-ratio or fold change (M). (XLS 326 kb) Additional file 3: Figure S1. Comparison of relative mRNA expression from biological replicate r1 and r2 using quantitative RT-PCR (qRT-PCR) and RNA-Seq of various genes show that both methods yield similar results in most cases. Note that all graphs have different scaling and show relative expression levels for qRT-PCR and RPKM values for RNA-Seq data. **A**. Comparison of quantitative RT-PCR and RNAseq data of DEGs involved in iron metabolism. The two biological replicates are shown in RPKM values (RNAseq) and relative expression levels (qRT-PCR). The middle column shows the obtained corrected RPKM values from both replicates. **B**. Comparison of quantitative RT-PCR and RNAseq data of DEGs from the Hexadehydroastechrome (HAS) cluster. Columns as mentioned in A. **C**. Comparison of quantitative RT-PCR and RNAseq data of some DEGs discussed on different paragraphs in the text. Columns as mentioned in A. All mRNA levels were measured by qRT-PCR with primers specific for the corresponding gene (Additional file 5: Table S4), and normalization with *akuA* primers were carried out. The delta CT method including efficiencies was used for quantification. (ZIP 940 kb) Additional file 4: Table S3. List of differential expressed genes (DEGs) related to the significantly enriched categories from the FunCat and GO functional analysis. A *p*-value of 0.05 was used for the enrichment method. For FunCat the first and second level is shown and when appropriate even higher levels. (XLS 28782 kb) Additional file 5: Table S4. Oligonucleotides used for quantitative RT-PCR. (XLS 43 kb)

## Abbreviations

DEG: Differential expressed gene
FunCat: Functional catalogue
GO: Gene ontology
qRT-PCR: Quantitative real time PCR
HAS: Hexadehydroastechrome cluster
IPA: Invasive pulmonary aspergillosis
IA: Invasive aspergillosis
