# Supplementary figures and images for: RNAseq analysis of Aspergillus fumigatus in blood reveals a just wait and see resting stage behavior
Source: BMC Genomics. 2015 Aug 27;16(1):640. doi: 10.1186/s12864-015-1853-1 (PMC4551469; doi:10.1186/s12864-015-1853-1)

Figure S1A

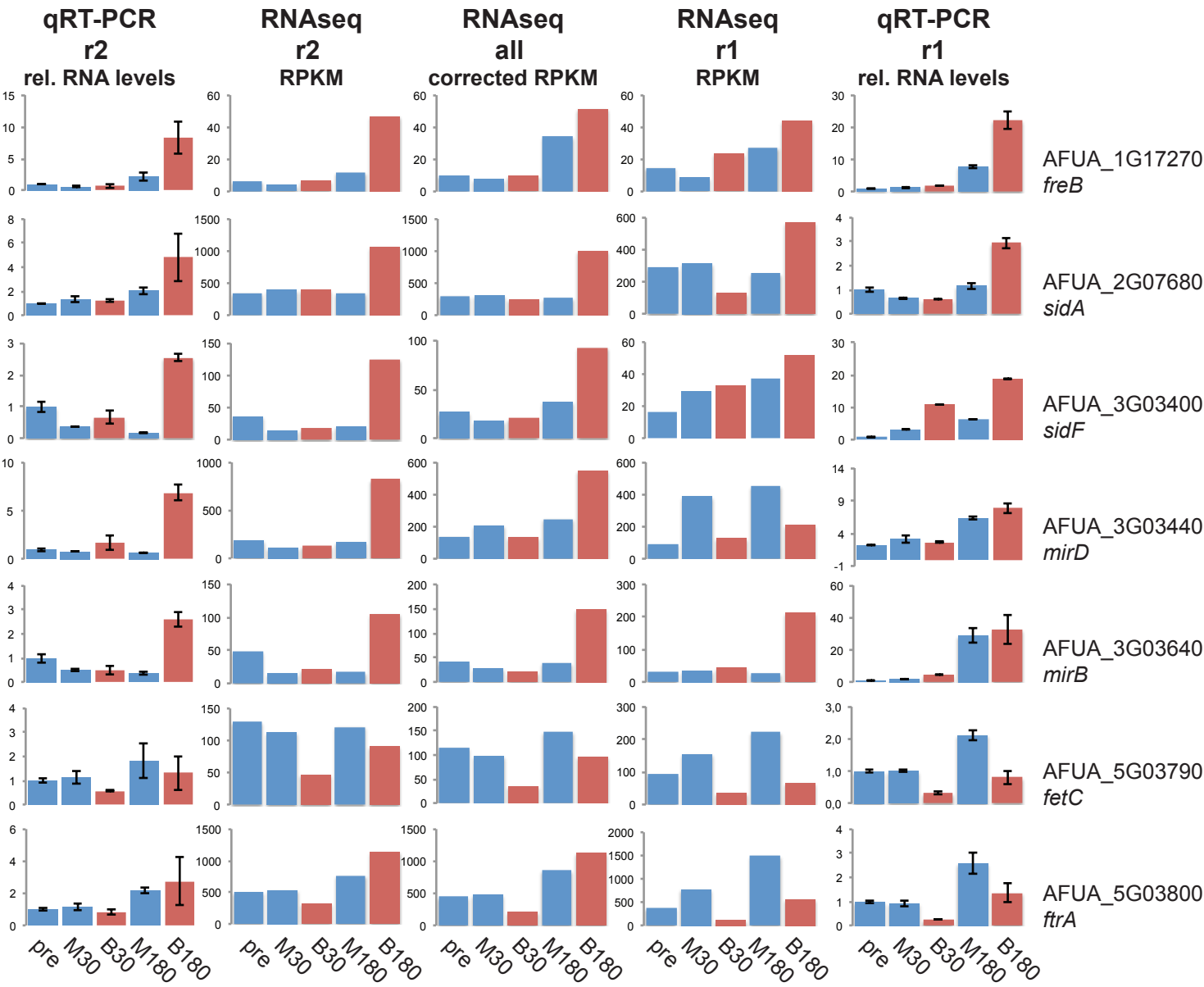

Supplement: Additional file 3: Figure S1. — Comparison of relative mRNA expression from biological replicate r1 and r2 using quantitative RT-PCR (qRT-PCR) and RNA-Seq of various genes show that both methods yield similar results in most cases. Note that all graphs have different scaling and show relative expression levels for qRT-PCR and RPKM values for RNA-Seq data. A. Comparison of quantitative RT-PCR and RNAseq data of DEGs involved in iron metabolism. The two biological replicates are shown in RPKM values (RNAseq) and relative expression levels (qRT-PCR). The middle column shows the obtained corrected RPKM values from both replicates. B. Comparison of quantitative RT-PCR and RNAseq data of DEGs from the Hexadehydroastechrome (HAS) cluster. Columns as mentioned in A. C. Comparison of quantitative RT-PCR and RNAseq data of some DEGs discussed on different paragraphs in the text. Columns as mentioned in A. All mRNA levels were measured by qRT-PCR with primers specific for the corresponding gene (Additional file 5: Table S4), and normalization with akuA primers were carried out. The delta CT method including efficiencies was used for quantification. (ZIP 940 kb) [file 12864_2015_1853_MOESM3_ESM.zip › FigS1A.pdf]

**B**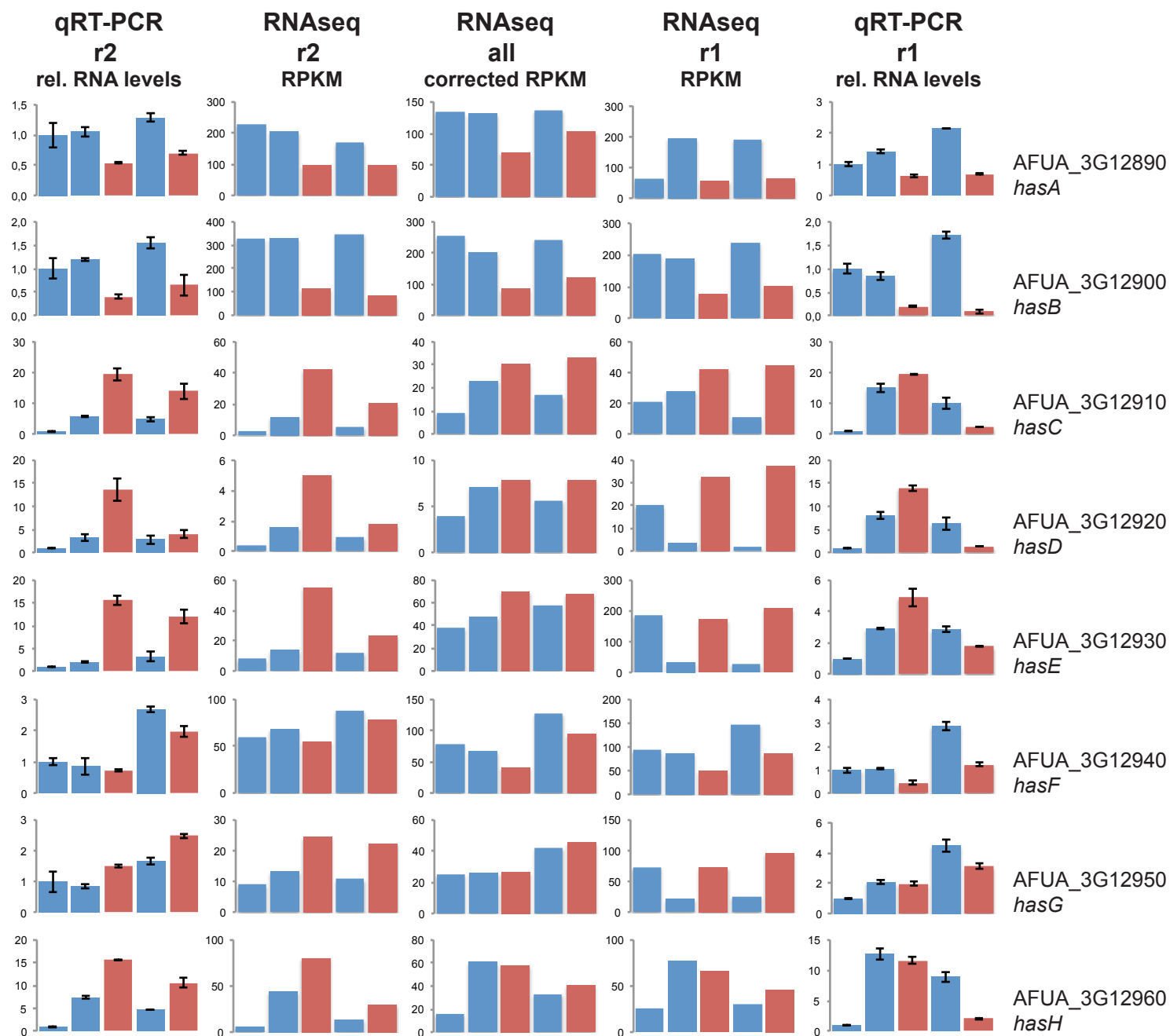**C**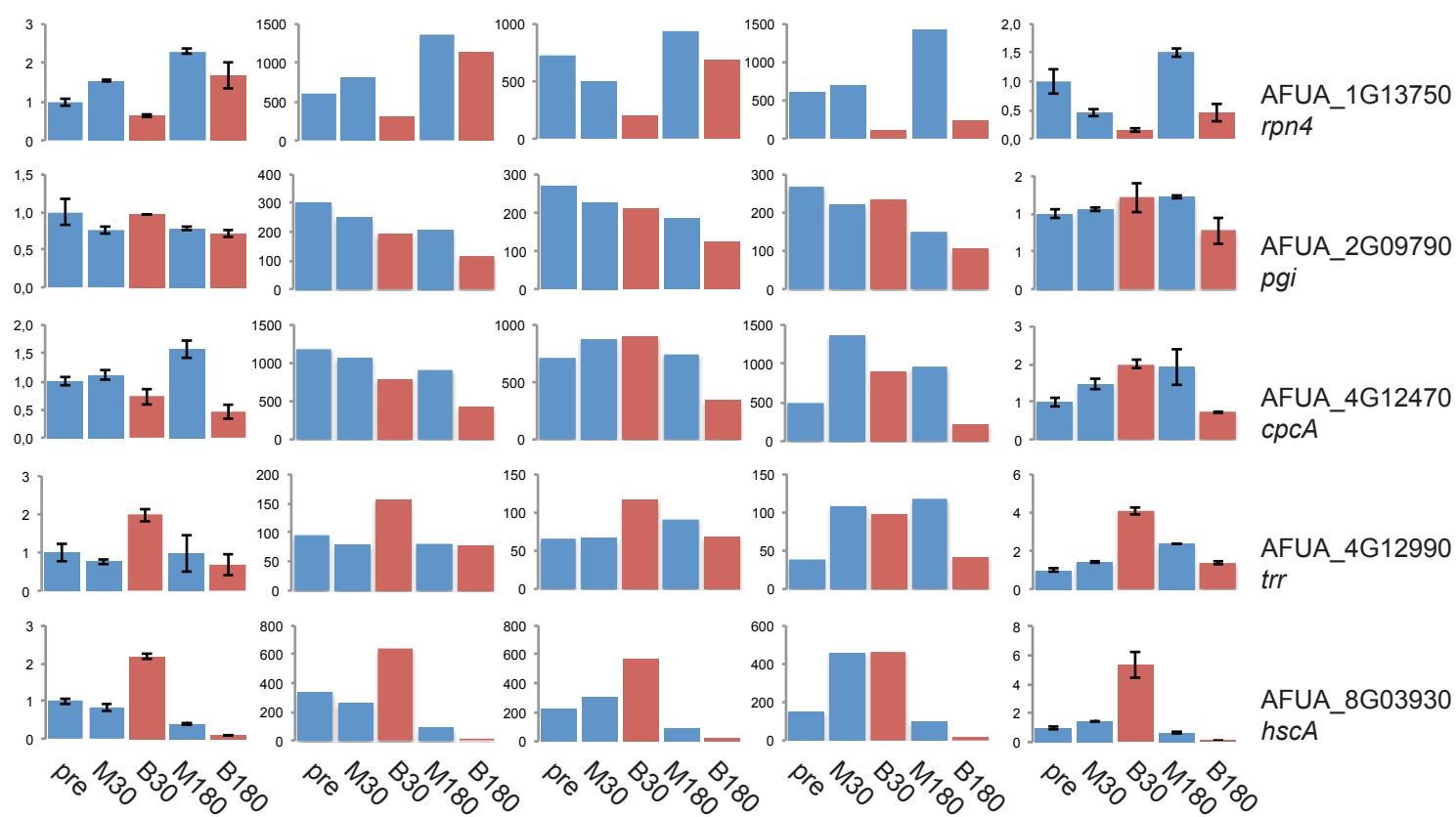

Supplement: Additional file 3: Figure S1. — Comparison of relative mRNA expression from biological replicate r1 and r2 using quantitative RT-PCR (qRT-PCR) and RNA-Seq of various genes show that both methods yield similar results in most cases. Note that all graphs have different scaling and show relative expression levels for qRT-PCR and RPKM values for RNA-Seq data. A. Comparison of quantitative RT-PCR and RNAseq data of DEGs involved in iron metabolism. The two biological replicates are shown in RPKM values (RNAseq) and relative expression levels (qRT-PCR). The middle column shows the obtained corrected RPKM values from both replicates. B. Comparison of quantitative RT-PCR and RNAseq data of DEGs from the Hexadehydroastechrome (HAS) cluster. Columns as mentioned in A. C. Comparison of quantitative RT-PCR and RNAseq data of some DEGs discussed on different paragraphs in the text. Columns as mentioned in A. All mRNA levels were measured by qRT-PCR with primers specific for the corresponding gene (Additional file 5: Table S4), and normalization with akuA primers were carried out. The delta CT method including efficiencies was used for quantification. (ZIP 940 kb) [file 12864_2015_1853_MOESM3_ESM.zip › FigS1BC.pdf]
